# Problems to affect long-term survival for breast cancer patients: An observational study of subsequent lung/bronchus malignancies

**DOI:** 10.1097/MD.0000000000012603

**Published:** 2018-09-28

**Authors:** Jieqiong Liu, Zheyu Hu, Yuhua Feng, Shan Zeng, Meizuo Zhong

**Affiliations:** a Department of Oncology, Xiangya Hospital, Central South University; b Hunan Cancer Hospital/The Affiliated Cancer Hospital of Xiangya, School of Medicine, Central South University, Changsha, People's Republic of China.

**Keywords:** breast cancer survivors, death cause, epidemiology and end results (SEER) database, risk factors, subsequent lung/bronchus malignancies

## Abstract

The overall survival of breast cancer (BC) patients increased significantly for decades; however, their long-term survival was seriously impaired by subsequent malignancies. This study aimed to investigate the risk factors of subsequent lung / bronchus primary malignancies among BC survivors.

A total of 535,941 BC female survivors diagnosed were identified by using SEER^∗^Stat database in 1973 to 2014. Among them, 9398 had subsequent lung/bronchus malignancies. Clinico-pathological risk factors were evaluated for the development of subsequent lung/bronchus cancer. The main measures were the incidence and risk factors of subsequent lung/bronchus primaries. Logistic regression analysis and survival analysis were performed.

Overall, among 535,941 BC survivors, 73,394 (13.69%) patients with subsequent primaries were identified from 1973 to 2014. The overall medium second tumor-free time was 72 months. Estrogen receptor (ER)-positive, progesterone receptor (PR)-positive, human epidermal growth factor receptor-2 (HER2)-positive, radiotherapy, and surgery treatment were protective factors against overall subsequent malignancies, whereas HER2/hormone receptor (HR) subtype triple negative, increasing tumor size, low differentiation grade, and high TNM stage were risk factors associated with overall subsequent malignancies. Surgical implantation reconstruction was risk factor for lung/bronchus cancer. Even though BC patients had a favorite 5-year survival, their long-term survival was affected by subsequent malignancies, especially for lung/bronchus cancer with high mortality.

Nearly 13% BC survivors suffered from subsequent malignancies. Increased risk was related to HER2/HR triple negative and advanced TNM stages. Radiotherapy and surgery were protective factors. Our findings may inform the subsequent cancer counseling of female BC survivors.

## Abstract 2

Supplemental Digital Content is available in the text

## 1 Introduction

Breast cancer (BC) is the most prevalent cancer among females.^[1]^ Due to the greater use of mammography screening and menopausal hormones, the incidence rate of BC increased rapidly in 1980s and1990s.^[2,3]^ After 2002, with the decreased use of menopausal hormones,^[4]^ BC rates decreased, especially in Caucasian women and for hormone receptor (HR)-positive BC.^[5,6]^ After 2010, BC incidence began to increase for women aged 60 years or older.^[2]^ BC is the leading cause of cancer-related death in females.^[7]^ In USA, the overall 5-year relative survival rate was 75.1% from 1975 to 1977, and has improved to 90.0% for 2001 through 2007.^[8]^ The death rate of BC has been declining since the early1990s in USA.^[3,9]^ Improvements in BC survival over decades are attributed to the prevalence of population-based screening using mammography and the systematic use of adjuvant therapies.^[10]^ However, even with treatment improvements, subsequent malignancies after BCs are still problematic and lead to poor long-term prognosis and death. Some researchers have focused on treatment-related subsequent malignancies.^[11–13]^ In estrogen receptor (ER)-positive BCs, Tamoxifen is widely used. However, this group of patients has a significantly higher risk of developing a subsequent endometrial cancer.^[14,15]^ However, based on a population study from Taiwan, antiestrogen use in BC patients is associated with a reduced risk of subsequent lung cancer.^[16]^ Another example is about therapy-related acute promyelocytic leukemia (tAPL). tAPL has been found to develop usually within 3 years after a primary neoplasm, especially BC, treated, in particular, with topoisoemerase II-targeted drugs.^[17]^ In addition, radiotherapy for primary BC could induce angiosarcoma^[18]^ and lung cancer.^[19,20]^ Older radiotherapy techniques are suggested to induce ipsilateral lung cancer, but no clear evidence has been demonstrated for modern radiotherapy techniques.^[21]^

Breast cancer survivors are at risk of subsequent lung cancer. The 5-year survival rate of primary lung/bronchus cancer was only 18.1% based on 2010 to 2014 Surveillance, Epidemiology, and End Results Program (SEER) surveillance database. Lung cancer patients with a previous malignancy have a similar survival rate with patients diagnosed as lung cancer as the first malignancy.^[22]^ The median overall survival times for localized, regional, and distant BC subsequent lung cancer patients are 5.1 years, 1.9 years, and 5.8 months, respectively.^[23]^ Therefore, even BC patients have a favorable 5-year survival rate; their long-term survival might be adversely affected by subsequent lung/bronchus cancers.

The objective of this study was to provide comprehensive information about the prevalence of subsequent malignancies after BC, to systematically analyze the risk factors, and to evaluate the impact of subsequent lung/bronchus malignancies on long-term survival of BC patients by using SEER research database.

## 2 Methods

### 2.1 Database and cohort definition

The SEER^∗^Stat database (8.3.4) was used as the data source in the present study. The SEER database is approved by NIH Ethics Program (both the NIH Ethics Office and individual ethics program in each Institute and Center). All procedures performed in studies involving human participants were in accordance with the ethical standards of the institutional and/or National Research Committee, and with the 1964 Helsinki declaration and its later amendments or comparable ethical standards. SEER database NIH Ethics Program was responsible for the informed consent (both the NIH Ethics Office and individual ethics program in each Institute and Center). Female patients firstly diagnosed with primary breast malignant tumors (site: C50.0-C50.9; World Health Organization [WHO] classification: ductal carcinomas, lobular carcinoma, special subtypes of invasive carcinoma, race invasive carcinoma) from 1973 to 2013, with positive histology diagnostic confirmation (n = 847,542) were identified in the SEER 18 Regs Research Data+ Hurricane Katrina Impacted Louisiana Cases, Nov 2015 Sub (1973–2013 varying) incidence database. Patients with 2 or more malignancies were also extracted from SEER^∗^Stat database. Subsequent malignancies were identified by matching patient ID. Patients with unspecified subsequent tumors (n = 29,639), or having subsequent tumors within 6 months after the diagnosis of the primary BC (n = 22,028), or having subsequent breast tumors in the same laterality with the first primary BC (n = 6527) were excluded from this study.

### 2.2 Measurements and candidate risk factors

The primary measurement was the incidence of subsequent malignancies among BC survivors. The subsequent measurement was the subsequent cancer-specific mortality of BC survivors. Candidate risk factors included age, race, HR/human epidermal growth factor receptor-2 (HER2) status, tumor size, differentiation grade, histology type, laterality, American Joint Committee on Cancer (AJCC) TNM stage, radiotherapy, and surgery subtypes. For demographic and clinico-pathological candidates, numeric variables were summarized as the mean (standard deviation) and median (interquartile range). Categorical variables were reported as counts (percentage). An analysis of variance was used to compare continuous variables with symmetric distributions across comparing subgroups. Chi-square tests or Fisher exact tests (n < 5) were used to compare categorical variables between clinical/pathological subgroups (race, stage, HR/HER2 subtypes, etc).

### 2.3 Multiple primary standardized incidence ratio calculation by using SEER^∗^Stat

For multiple subsequent cancers, multiple primary standardized incidence ratio (MP-SIR) was calculated by using observed secondary events versus expected secondary event based on SEER 9 registry rate file. The analysis cohort was patients with primary BC as the first primary malignancy ({Site and Morphology. Site recode B ICD-O-3/WHO2008} = ’ Breast’; {Multiple Primary Fields. Sequence number} = ’1st of 2 or more primaries’). Subsequent lung/bronchus cancer was set as events. The 1, 5, and 10-year and overall MP-SIRs for subsequent lung/bronchus postprimary BC were extracted from January, 1973 to December, 2014.

### 2.4 Survival analysis and Cox regression model

Survival analysis was performed for right-censored datasets. Because patients who were lost to follow-up because of definite reasons should be excluded from the survival test, so patients who died before the existence of secondary malignancies were excluded from the sample in this study. The Kaplan-Meier method was used to plot the survival distributions against subsequent malignancies or cancer-specific death, and the log-rank test was used to assess differences in survival experience among the clinical/pathological subgroups. To identify the risk factors for subsequent malignancies, the Cox proportional-hazards regression was performed to estimate the hazard ratio. A receiver-operating characteristic (ROC) curve and the area under the curve (AUC) measure the efficiency of age in predicting subsequent malignancies. All tests of hypotheses were 2-tailed and conducted at a significance level of .05. Statistical analyses were conducted using SAS 9.4.

## 3 Results

### 3.1 Incidence of subsequent malignancies

**Figure 1:**
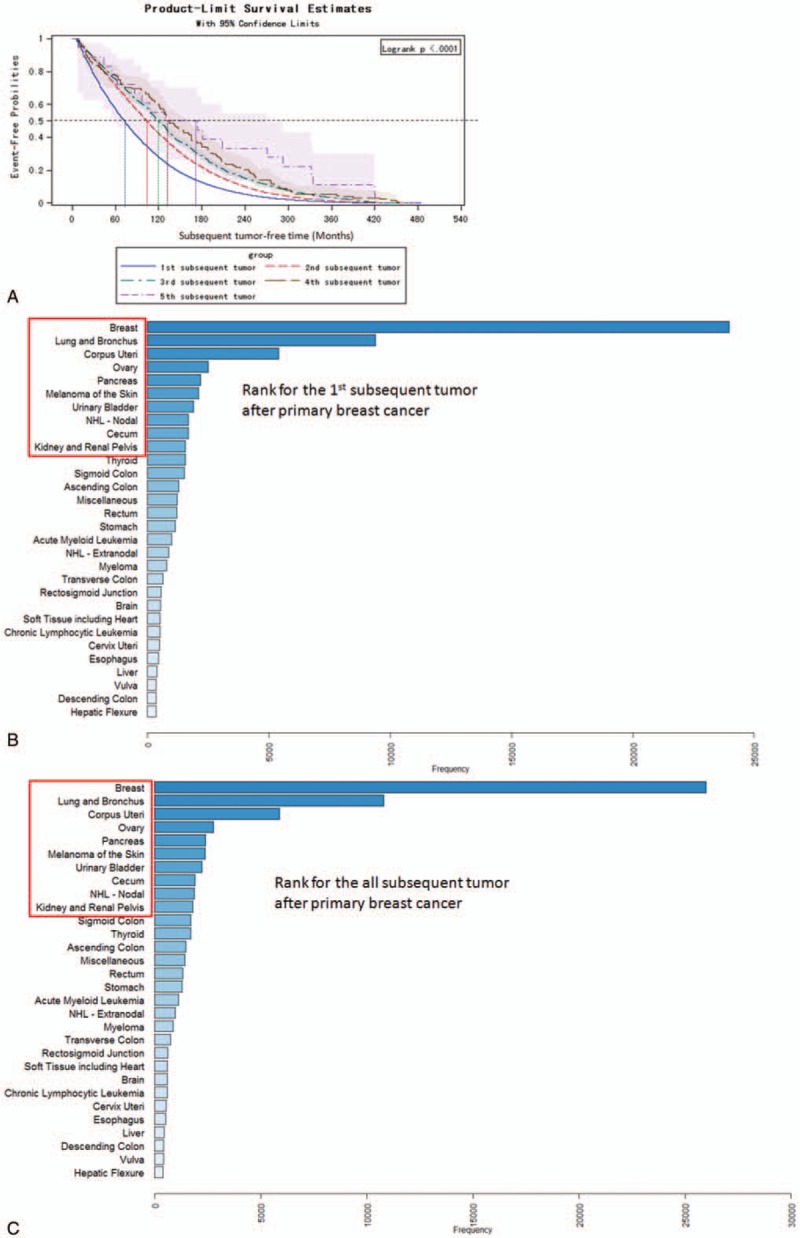
Median occurrence time and rank of subsequent malignancies after BC. (A) KM curves for subsequent malignancies incidence stratified by the sequences of subsequent malignancies. (B) Sites spectrum of the first subsequent malignancies in BC patients. (C) Sites spectrum of the all subsequent malignancies in BC patients. BC = breast cancer.

Of 847,542 primary BC patients, 715,954 patients had no subsequent malignancies and 253,407 patients died before having a subsequent malignancy. In 535,941 BC survivors (patients alive before having a definite subsequent malignancy), 73,394 (13.69%) patients had definite subsequent malignancies. Also, 65,989 (12.31%), 6616 (1.23%), 716 (0.13%), 64 (0.01%), and 9 (0.0017%) patients had 1, 2, 3, 4, and 5 subsequent tumors, respectively (eFig. 1). The median subsequent tumor-free time for the first to fifth subsequent malignancies was 72, 104, 120, 133.5, and 151 months, respectively (Fig. 1A, *P* < .0001).The site spectrum of subsequent malignancies indicated that breast, lung/bronchus, and ovary/uteri were the most prevalent sites (Fig. 1B and C). The top 3 first and all subsequent malignancies were contralateral BC (CBC), lung/bronchus cancer, and uteri/ovary cancer. In all, 10,789 patients developed subsequent lung/bronchus cancer, and 9398 patients with lung/bronchus cancer as the first subsequent malignancy after primary BC. The median subsequent tumor-free time for subsequent lung/bronchus malignancies was about 6.5 years (78 months; Table 1). The 1, 5, 10, and 10+-year MP-SIR for subsequent lung/bronchus cancer in initial BC patients were 4.13, 5.18, 5.14, and 5.40, respectively, in 1973 to 2014. These statistics suggested a significantly higher incidence of subsequent lung/bronchus cancer in BC survivors than those in general populations.

**Table 1:**
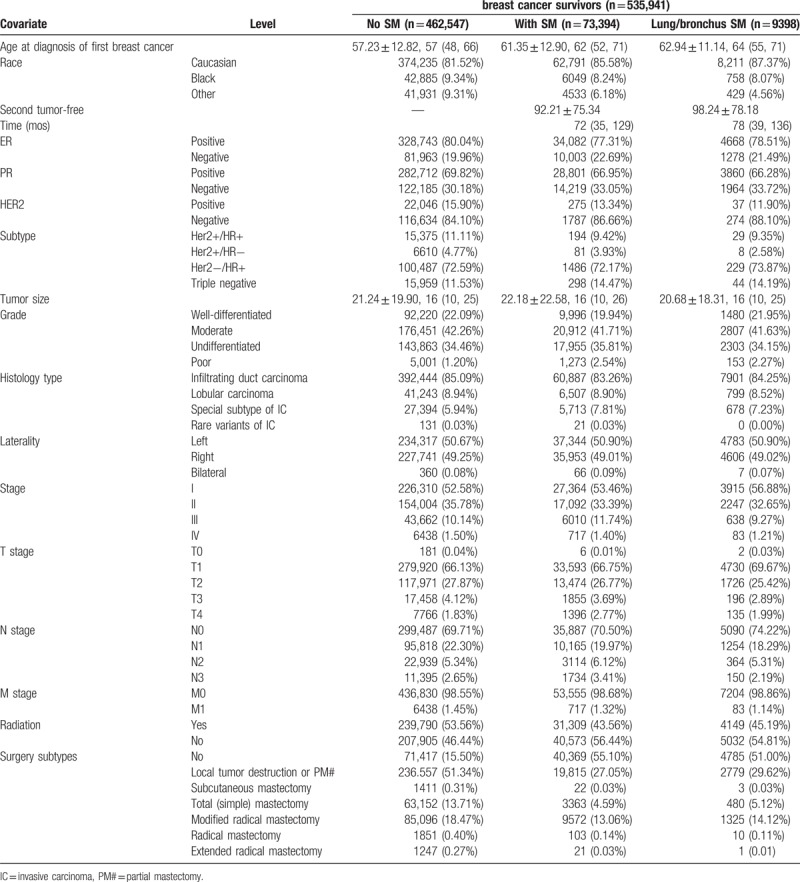
Demographics and clinical features of breast cancer survivors with/without subsequent malignancies (SMs) at lung/bronchus.

### 3.2 Trend of subsequent malignancies

**Figure 2:**
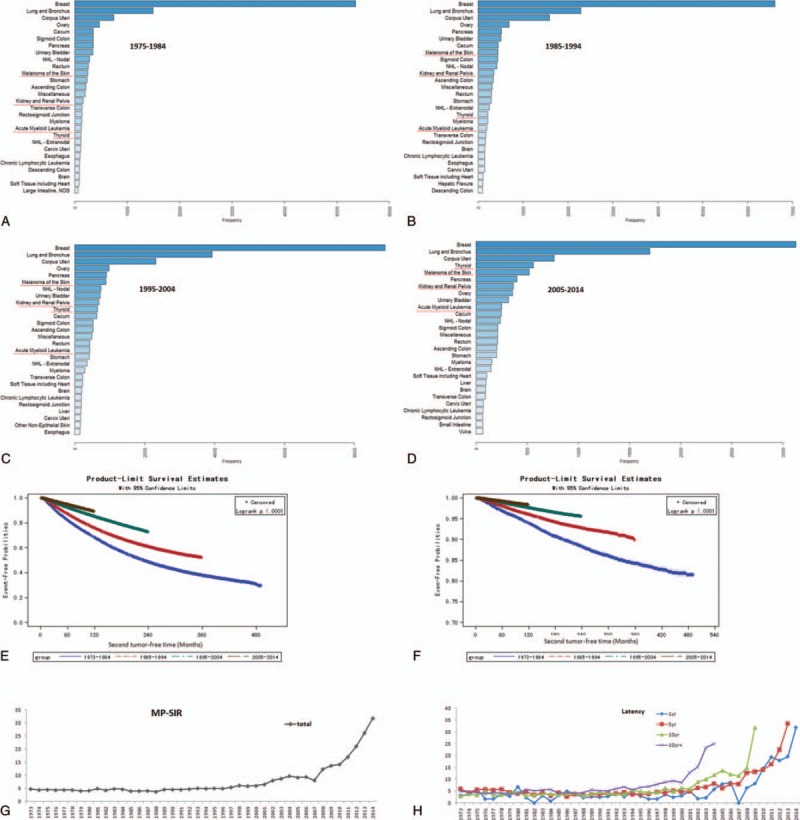
Time trend for the subsequent lung/bronchus malignancies in BC survivors. (A–D) Sites spectrum of subsequent malignancies in 1973 to 1984 (A), 1985 to 1994 (B), 1995 to 2004 (C), and 2005 to 2014 (D). (E) Event-free probabilities for subsequent malignancies at all sites in BC survivors. (F) Event-free probabilities for subsequent malignancies at lung/bronchus in BC survivors. (G) Total MP-SIR trend of subsequent lung/bronchus malignancies among BC survivors from1973 to 2014. (H) Time trend of MP-SIR with 1, 5, and 10-year latency for subsequent lung/bronchus malignancies among BC survivors from 1973 to 2014. BC = breast cancer.

Interestingly, the site spectrum of other subsequent malignancies varied during past 4 decades. As demonstrated in Fig. 2A to D, thyroid carcinoma increased significantly from the 20th place in 1973 to 1984, to 4th place in 2005 to 2013. Apart from thyroid cancer, melanoma of the skin, kidney and renal pelvis cancer, and acute myeloid leukemia also increased significantly, especially in recent 2 decades. However, from 1973 to 2014, lung/bronchus cancer was always the top subsequent cancer except BC. Kaplan-Meier curves were adopted to compare the event-free rate against subsequent malignancies. According to the diagnosis year of first BC, patients were divided into 4 subgroups: 1973 to 1984, 1985 to 1994, 1995 to 2004, and 2005 to 2014. As shown in Fig. 2E to F and eTable 1, 5, 10, and 15-year event-free rates against subsequent malignancies or lung/bronchus tumors increased significantly from 1973 to 1983, to 2004 to 2013. Compared with BC patients diagnosed in 1973 to 1984, the hazards of subsequent malignancies or lung/bronchus tumors were significantly lower in patients diagnosed in 1985 to 1994, 1995 to 2004, and 2005 to 2014, with hazard ratio of 0.685 (95% confidence interval [CI] 0.669, 0.701), 0.419 (95% CI 0.409, 0.429), and 0.284 (95% CI 0.277, 0.292), respectively, for all subsequent tumors, and 0.588 (95% CI 0.547, 0.632), 0.357 (95% CI 0.333, 0.383), and 0.250 (95% CI 0.231, 0.271), respectively (eTable 2).

By calculating MP-SIR, we found that the incidence of lung/bronchus cancer in primary BC patients was about 5-fold higher, compared with general population (Fig. 2G). From 2000 to 2014, the MP-SIR began to strikingly increase to 31.83. As shown in Fig. 2H, for BC patients who were diagnosed in 2004, the 1, 5, 10, and 10+-year MP-SIR of subsequent lung/bronchus cancer were 5.79, 8.15, 11.85, and 25, respectively, which were much higher than patients diagnosed in 1994. In BC patients diagnosed in 1994, the 1, 5, 10, and 10+-year MP-SIR of subsequent lung/bronchus cancer were 4.31, 3.65, 5.33, and 5.56, respectively.

### 3.3 Clinico-pathological features and risk factors of subsequent malignancies

Patients with subsequent malignancies were older than patients without subsequent malignancies (61.35 ± 12.90 vs 57.23 ± 12.82; Table 1). But ROC curve showed minimal value of age in predicting subsequent malignancies (AUC 0.5283; eFig. 2A). By plotting the amount of patients having subsequent tumors at their age of BC diagnosis, the maximum amount of patients existed at 65-year old (eFig. 2B), which lagged 3 years compared with median diagnosis age of BC (62-year-old). Females aged 55 to 64 years have the highest rate to be diagnosed with BC. To avoid the influence of the diagnosis age at first BC, we further plotted the relative rate of patients having subsequent malignancies at their diagnose year of the first BC. As shown in eFig. 2C, more than 20% of patients diagnosed in their 70s had subsequent malignancies. Patients diagnosed at 78 years had the highest rate to have subsequent malignancies. After 78, the rate decreased, suggesting that BC survivors diagnosed in their 80s, or who were older, were less likely to have subsequent malignancies.

For other clinico-pathological features, patients of white race, tumors with ER, progesterone receptor (PR), HER2, triple negative, poorly differentiated tumor, special subtype of invasive carcinoma, T4/N2-3, patients without radiation and without surgery had higher incidence of subsequent malignancies (Table 1). Patients with subsequent lung/bronchus cancer had much higher all-cause mortality rate and lung cancer-specific mortality. To further evaluate the effect of clinico-pathological risk factors on subsequent malignancies, univariate Cox regression analysis was performed. As demonstrated in Table 2, HR/HER2 triple negative, poor differentiation grade, advanced TNM stage, and the lack of radiotherapy or surgery were significant risk factors associated with the subsequent malignancies after first primary BC. For subsequent lung/bronchus cancer, old-aged, T4, M1, and stage IV, surgery reconstruction with implantation was a significant risk factor; PR-positive, HER2-positive, radiation, and surgery were protective factors. Unlike previous reports about risk factors HR-positive and anticancer treatment-related subsequent malignancies (14-15,18), our findings suggested HR-positive and radiotherapy/surgery to be protective factors. The development of adjuvant treatment and surgery might prevent or delay the subsequent malignancies’ occurrence.^[24,25]^

**Table 2:**
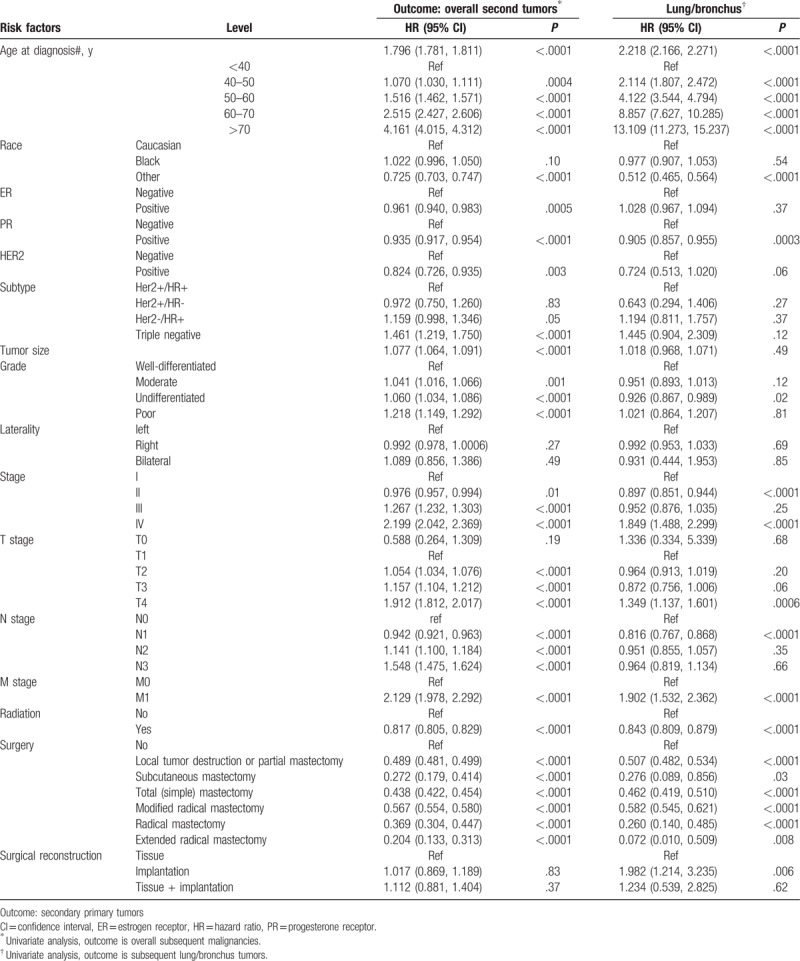
Risk factors for free of secondary primary tumors.

### 3.4 Time trend of radiation and surgery on subsequent lung/bronchus malignancies

Radiotherapy of BC patients exposed their lung under radiation. Exposure of the lung to radiotherapy depends on many factors, including the radiation source, treatment plan, technique, and fractionation.^[21]^ Older radiotherapy techniques were suggested to be associated with an elevated risk of developing lung cancer in the ipsilateral lung.^[21]^ As shown in Table 3, BC patients diagnosed in 1973 to 1984 with radiotherapy had a higher risk of developing subsequent lung/bronchus cancer, compared with the nonradiotherapy group (hazard ratio 1.647, 95% CI 1.451, 1.870). In recent decade, radiotherapy became a protective factor against subsequent lung/bronchus cancer (hazard ratio 0.836, 95% CI 0.767, P = 0.913). For surgical treatment, surgery was a protective factor against subsequent lung/bronchus cancer from 1973 to 2014. Surgical reconstruction emerged in 1998. In the first decade of use (1998–2004), implantation had a higher risk than tissue reconstruction (hazard ratio 2.479, 95% CI 1.301, 4.721). In 2005 to 2014, the risk of implantation versus tissue reconstruction decreased (hazard ratio 1.506, 95% CI 0.711, 3.188). In this study, information of chemotherapy was not included in SEER database.

**Table 3:**
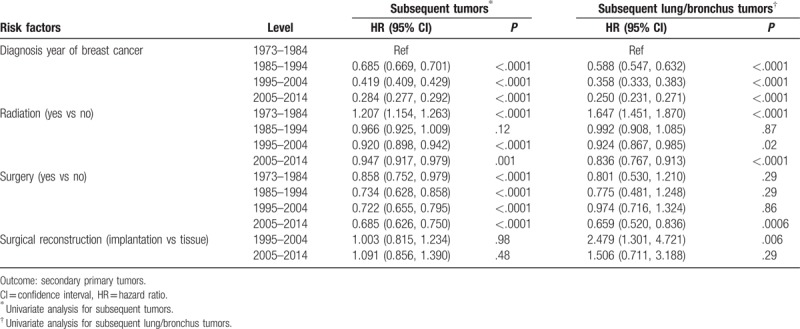
Risk factors for free of secondary primary tumors among patients firstly diagnosed as breast cancer in 1973 to 1984, 1985 to 1994, 1995 to 2004, and 2005 to 2014.

### 3.5 Mortality rate and death causes for BC patients with subsequent malignancies

**Figure 3:**
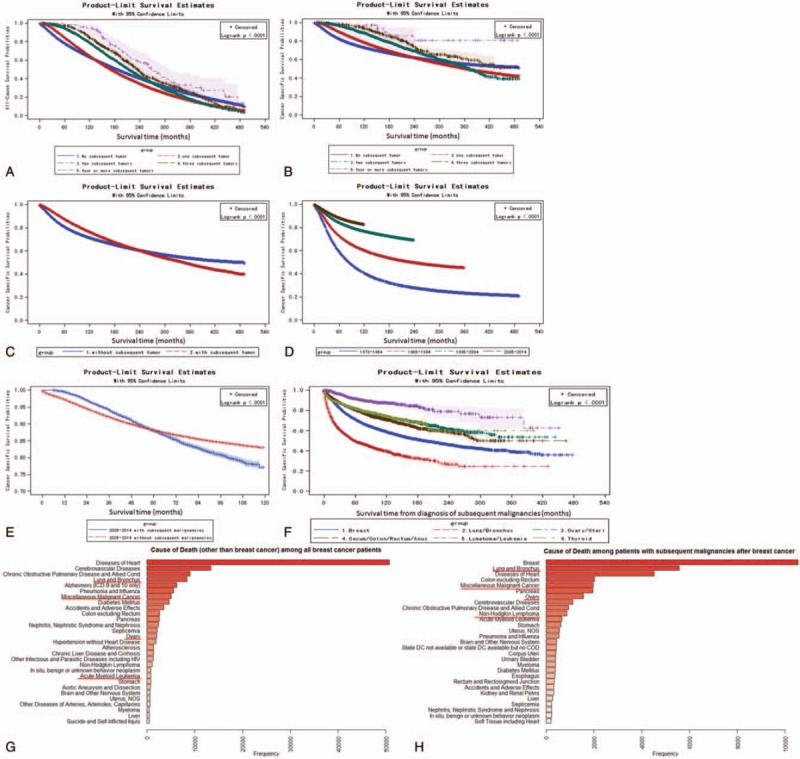
Mortality rate and death causes for BC patients with subsequent malignancies. (A, B) KM curves for all-cause mortality (A) and cancer-specific mortality (B) stratified by the sequence of subsequent malignancies. (C) KM curves for cancer-specific mortality stratified by with or without subsequent malignancies. (D) KM curves for cancer-specific mortality stratified by first BC diagnosis decades. (E) KM curves for cancer-specific mortality in 2004 to 2013 stratified by with or without subsequent malignancies. (F) KM curves for cancer-specific mortality stratified by sites of the subsequent malignancies. (G) Spectrum of death causes (except BC itself) among all BC patients included. (H) Spectrum of death causes among BC patients with subsequent malignancies. BC = breast cancer.

Patients with subsequent malignancies had significantly higher all-cause mortality and cancer-specific mortality than patients without subsequent malignancies (eTable 3). Though patients with subsequent malignancies had higher survival rate against both all-cause mortality and cancer-specific mortality than patients without subsequent malignancies within 10 years, their long-term survival rate was lower than patients without subsequent malignancies (Fig. 3A–C).The survival information were listed in eTables 4 and 5. In addition, the survival rate of BC patients varied during past 4 decades. As demonstrated in Fig. 3D, the 5-year survival rate increased from 0.5883 for patients diagnosed in 1973 to 1984, to 0.8837 for those diagnosed in 2005 to 2014 (eTable 5); also, 10 and 15-year survival rate increased for patients diagnosed in recent decades. Moreover, according to SEER database 2005 to 2014, even the 5-year survival rate of BC patients is about 88% in USA; their long-term survival is still problematic due to subsequent malignancies. The 9-year cancer-specific survival rate for patients with subsequent malignancies was 0.7878, which was significantly lower than the survival rate of 0.8362 for patients without subsequent malignancies (Fig. 3E, *P* < .0001; eTable 6).

To further investigate the survival rate after patients diagnosed with subsequent malignancies at different sites, life test was performed, and the observing start time was set at the occurrence of subsequent malignancies. KM curves showed that patients with lung/bronchus cancer had lowest survival rate, whereas patient with subsequent lymphoma/leukemia had the highest survival rate (Fig. 3F). The 10-year survival rate for patients with subsequent BC, lung/bronchus cancer, ovary/uteri cancer, cecum/colon/rectum/anus cancer, lymphoma/leukemia, and thyroid cancer were 0.5881, 0.3954, 0.7317, 0.7072, 0.8747, and 0.7421, respectively (eTable 7). Among all BC patients, BC is the leading cause of death. Except BC, heart disease was the leading cause of death and lung/bronchus cancer was the fourth leading cause of death (Fig. 3G). In patients with subsequent malignancies, BC was still the leading cause of death, and lung/bronchus cancer was the second leading cause of mortality (Fig. 3H).

## 4 Discussion

Lung/bronchus cancer was always the top subsequent malignancies except BC (Fig. 2A–D). MP-SIR data indicated an increase of subsequent lung/bronchus malignancies in BC patients in recent decade (Fig. 2G–H). However, survival analysis indicated that the subsequent lung/bronchus malignancies event-free probabilities decreased in recent decade compared with 1973 to 1984 (Fig. 2F). Such decrease was related to development of BC treatment techniques, including radiotherapy and surgical reconstruction (Table 3).

Though BC remained the leading of all cause of death, subsequent lung/bronchus cancer was considerably involved in the death events of BC patients, especially for those who suffered from subsequent lung cancer (Fig. 3G and H). Other cancer type including colon/rectum, ovary cancer, and non-Hodgkin lymphoma were also important cancerous causes of death. Interestingly, as for the site spectrum of subsequent malignancies, the rank of thyroid carcinoma increased significantly from the 20th place in 1973 to 1983, to the fourth place in 2004 to 2013. Such an increase is not striking. Based on SEER database, the annual percent change (APC) of thyroid cancer is about 7% for recent years, indicating the rate of thyroid cancer rising, surpassing rates of common cancers and becoming the third most common cancer in women by 2019.^[26]^ Apart from thyroid cancer, melanoma of the skin, kidney, and renal pelvis cancer, and acute myeloid leukemia also increased significantly, especially in recent 2 decades (Fig. 2A–D). By comparing the survival information of patients with subsequent malignancies at different sites, lung/bronchus cancer had the lowest survival curves (rates, Fig. 3F).

For BC, surgery is the primary treatment. About 20% to 45% of patients who receive mastectomy have breast reconstruction with implantation, tissue flap, or both.^[27,28]^ Surgical reconstruction/implantation is a significant risk factor related to subsequent lung cancer^[29]^ and anaplastic large cell lymphoma.^[30,31]^ Radiotherapy and chemotherapy also involve in the development of subsequent malignancies. Radiotherapy/chemotherapy is related to myelodysplasia and acute myeloid leukemia (tMDS/AML),^[11,32–34]^ lymphoma,^[35]^ soft tissue sarcoma^[36]^ and skin tumors,^[37]^ and so on. Other cancer related pathologies included virus infection,^[38]^ metabolism disorders,^[39]^ and genetic susceptibility,^[40]^ etc. However, in our study, we found that both radiation and surgery treatment were significant protective factors against subsequent malignancies (Table 2), especially in recent decade (2005–2014) (Table 3).

In our previous research, we have also found that breast cancer patients have great chance to bear secondary ovary /uterus cancer, colonrectal cancer and thyroid cancer.^[41]^*BRCA1/2* mutation is related to both breast cancer and ovary cancer.^[42]^ Breast cancer patients with a family history of breast or ovarian cancer also had an increased risk of subsequent leukemia.^[43]^ BC survivors with ER-negative/HER2-positive and triple-negative BC (TNBC) had a significantly increased risk of developing a second primary asynchronous CBC.^[24,44–45]^Table 2 indicated TNBC subtype to be risk factors, whereas ER-positive, PR-positive, and HER2-positive were protective factors. PR-positive and HER2-positive were also protective factors for subsequent lung/bronchus cancer, reflecting the improvement of postoperative adjuvant and endocrine therapy for BC patients.

## 5 Conclusions

Overall, our study provided comprehensive evaluation of the risk factors and survival outcome of subsequent malignancies in primary BC patients. Though the subsequent malignancies’ event-free probabilities increased tremendously in recent decade, MP-SIR of lung/bronchus cancer increased significantly from 2000. Further investigations should be initiated to establish reasonable surveillance strategies based on site-specific risk factors.

## Acknowledgments

This study used the SEER 18 Regs research database as the data source. The interpretation and reporting of these data are the sole responsibility of the authors. The authors acknowledge the efforts of the National Cancer Institute; the SEER Program tumor registry; and the Information Management Service Inc. for the creation and distribution of the SEER^∗^Stat database. No other funds were included in this study. None of the authors have competing interests.

## Author contributions

**Conceptualization:** Meizuo Zhong, Jieqiong Liu and Zheyu Hu.

**Data curation:** Jieqiong Liu, Zheyu Hu.

**Formal analysis:** Jieqiong Liu, Zheyu Hu.

**Investigation:** Jieqiong Liu, Zheyu Hu, Yuhua Feng, Shan Zeng, Meizuo Zhong.

## Supplementary Material

Supplemental Digital Content
