# Supplementary material for: Problems to affect long-term survival for breast cancer patients: An observational study of subsequent lung/bronchus malignancies
Source: Medicine (Baltimore). 2018 Sep 28;97(39):e12603. doi: 10.1097/MD.0000000000012603 (PMC6181575; doi:10.1097/MD.0000000000012603)
Supplement: Supplemental Digital Content [file medi-97-e12603-s001.doc]

eFigure 1. Data flow.

**
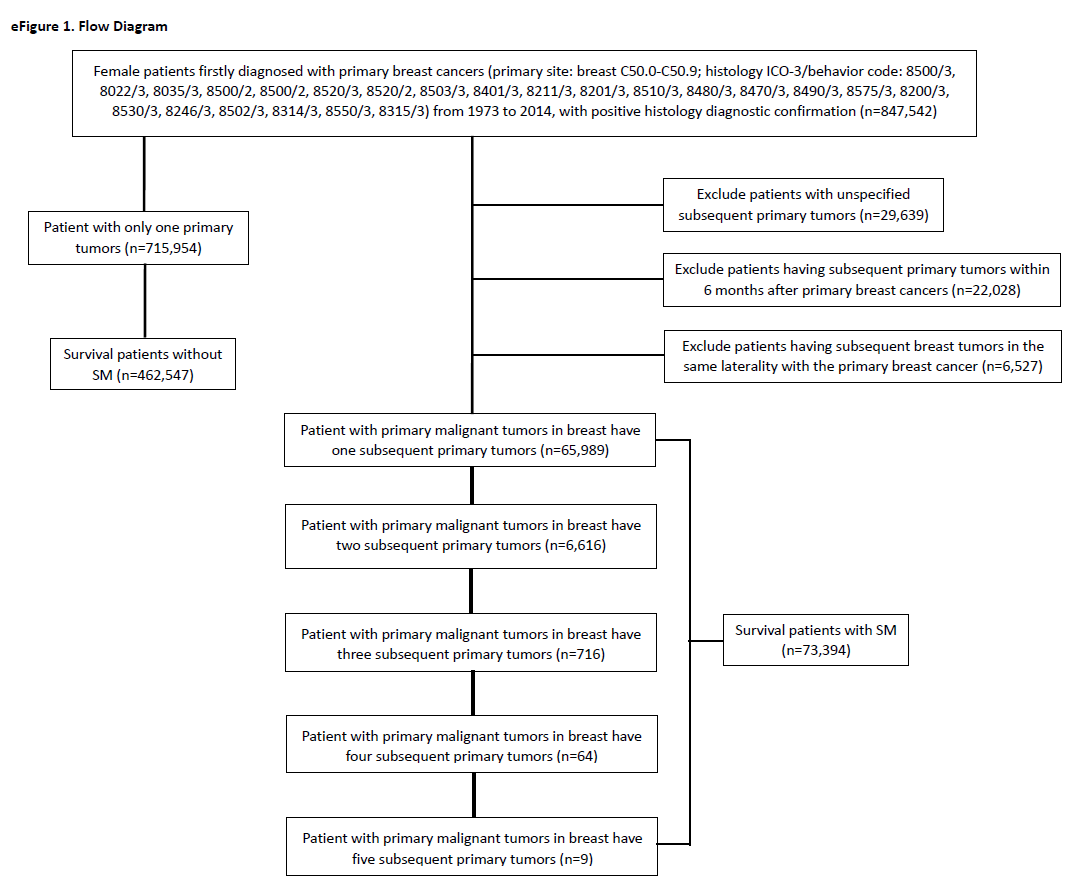
**

eFigure 2. Effect of age at diagnosis of BC on subsequent malignancy incidence.

A. ROC curve showed minimal value of age in predicting subsequent malignancies (AUC=0.5283).

B. Barplot showed the amount of patients having subsequent tumors at their age at BC diagnosis. The maximum amount of patients existed at 65-year old.

C. Barplot showed the relative rate of patients having subsequent malignancies at their diagnose year of the first BC. BC patients diagnosed at 78-year old had the highest rate to have subsequent malignancies.

**
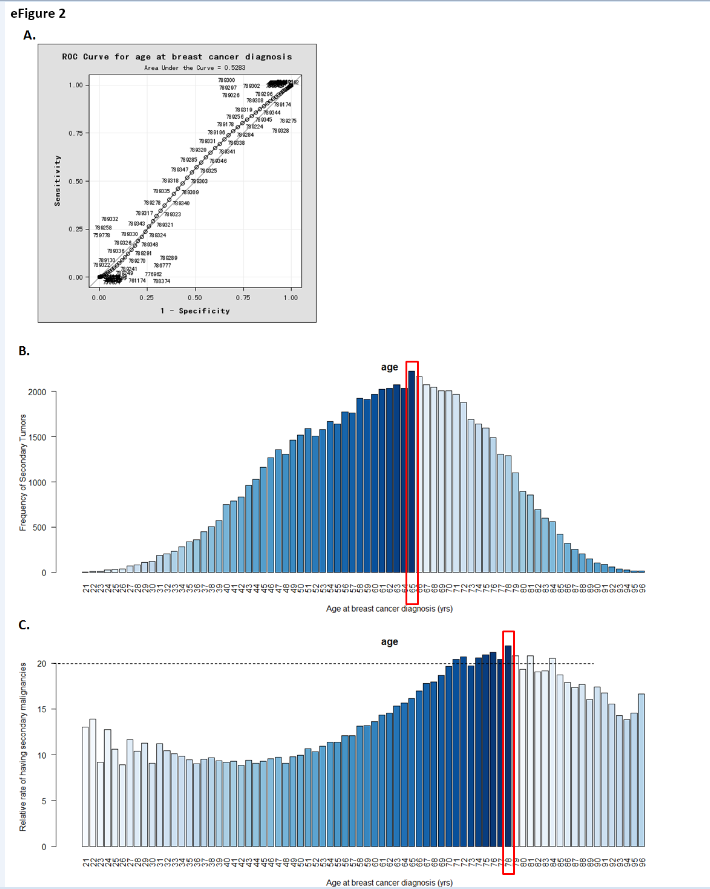
**

**eTable 1. Event-free rate information for all subsequent tumors stratified by first breast cancer diagnosis decades.**

|  | 1973-1984 | | | | 1985-1994 | | | | 1995-2004 | | | | 2005-2014 | | | |
| --- | --- | --- | --- | --- | --- | --- | --- | --- | --- | --- | --- | --- | --- | --- | --- | --- |
| Timelist (months) | Event Months | Survival probability | Number Failed | Number Left | Event Months | Survival probability | Number Failed | Number Left | Event Months | Survival probability | Number Failed | Number Left | Event Months | Survival probability | Number Failed | Number Left |
| 24 | 24 | 0.9311 | 1305 | 17609 | 24 | 0.9530 | 2022 | 40959 | 24 | 0.9723 | 4116 | 144181 | 24 | 0.9814 | 5017 | 231993 |
| 60 | 60 | 0.8212 | 3349 | 15441 | 60 | 0.8715 | 5518 | 37315 | 60 | 0.9233 | 11360 | 136037 | 60 | 0.9477 | 11241 | 125083 |
| 108 | 108 | 0.7085 | 5488 | 13204 | 108 | 0.7790 | 9472 | 33206 | 108 | 0.8650 | 19899 | 125481 | 108 | 0.9028 | 14620 | 19079 |
| 120 | 120 | 0.6833 | 5959 | 12713 | 120 | 0.7589 | 10328 | 32295 | 120 | 0.8512 | 21886 | 121013 | 119 |  | 14735 | 0 |
| 180 | 180 | 0.5707 | 8042 | 10487 | 180 | 0.6734 | 13946 | 28284 | 180 | 0.7870 | 27455 | 31011 | 119 |  | 14735 | 0 |
| 240 | 240 | 0.4860 | 9587 | 8783 | 240 | 0.6090 | 16620 | 24384 | 238 |  | 28543 | 0 | 119 |  | 14735 | 0 |
| 300 | 300 | 0.4259 | 10661 | 7490 | 300 | 0.5593 | 17902 | 7519 | 238 |  | 28543 | 0 | 119 |  | 14735 | 0 |
| 360 | 360 | 0.3796 | 11460 | 6315 | 357 |  | 18138 | 0 | 238 |  | 28543 | 0 | 119 |  | 14735 | 0 |
| 420 | 420 | 0.3447 | 11862 | 2288 | 357 |  | 18138 | 0 | 238 |  | 28543 | 0 | 119 |  | 14735 | 0 |
| 480 | 479 | 0.3080 | 11974 | 136 | 357 |  | 18138 | 0 | 238 |  | 28543 | 0 | 119 |  | 14735 | 0 |
| 500 | 484 |  | 11978 | 0 | 357 |  | 18138 | 0 | 238 |  | 28543 | 0 | 119 |  | 14735 | 0 |

**eTable 2**. Mortality rate of breast cancer patients with all subsequent cancers and subsequent cancers at lung / bronchus.

| Covariate | level | No subsequent cancers  (n=715,954) | Subsequent cancers  (n=73,394) | *p-value |  | Subsequent Lung/Bronchus  cancer (n=9,398) | #p-value |
| --- | --- | --- | --- | --- | --- | --- | --- |
| Survival months |  | 90.67 ± 80.46  69 (29, 131) | 149.14 ± 95.61  132 (75, 204) | <0.0001 |  | 123.91 ± 84.67  106 (59, 166) | <0.0001 |
|  |  |  |  |  |  |  |  |
| Second tumor-free time (months) |  |  | 92.21 ± 75.34  72 (35, 129) |  |  | 98.24 ± 78.18  78 (39, 136) |  |
|  |  |  |  |  |  |  |  |
| All-cause mortality | Alive | 462,547 (64.61%) | 29,212 (39.80%) | <0.0001 |  | 1,932 (20.56%) | <0.0001 |
|  | Death | 253,407 (35.39%) | 44,182 (60.20%) |  |  | 7,466 (79.44%) |  |
|  |  |  |  |  |  |  |  |
| Primary Breast  Cancer-specific death | Alive | 462,547 (77.85%) | 29,212 (70.45%) | <0.0001 |  | 1,932 (56.16%) | <0.0001 |
|  | Death | 131,606 (22.15%) | 12,253 (29.55%) |  |  | 1,508 (43.84%) |  |

Note: *p-values were calculated by comparing patients with subsequent cancers to patients without subsequent cancers. #p-values were calculated by comparing patients with subsequent lung / bronchus cancers with patients without subsequent cancers.

eTable 3. Survival rate information for all-cause mortality (A) and cancer-specific mortality (B) stratified by the sequence of secondary malignancies.

A.

|  | No secondary tumor | | | | One subsequent tumor | | | | Two subsequent tumors | | | | Three subsequent tumors | | | | Four or more subsequent tumors | | | |
| --- | --- | --- | --- | --- | --- | --- | --- | --- | --- | --- | --- | --- | --- | --- | --- | --- | --- | --- | --- | --- |
| Timelist (months) | Event Months | Survival probability | Number Failed | Number Left | Event Months | Survival probability | Number Failed | Number Left | Event Months | Survival probability | Number Failed | Number Left | Event Months | Survival probability | Number Failed | Number Left | Event Months | Survival probability | Event Months | Survival probability |
| 24 | 24 | 0.9054 | 63219 | 563846 | 24 | 0.9645 | 2334 | 63176 | 24 | 0.9932 | 45 | 6560 | 24 | 0.9972 | 2 | 714 | 0 | 1.0000 | 0 | 73 |
| 60 | 60 | 0.7806 | 133934 | 391303 | 60 | 0.8419 | 10224 | 52936 | 60 | 0.9457 | 357 | 6178 | 60 | 0.9720 | 20 | 693 | 57 | 0.9863 | 1 | 72 |
| 120 | 120 | 0.6320 | 195511 | 203281 | 120 | 0.6429 | 21986 | 34799 | 120 | 0.7876 | 1360 | 4802 | 120 | 0.8419 | 111 | 578 | 113 | 0.9450 | 4 | 68 |
| 180 | 180 | 0.5095 | 225387 | 88436 | 180 | 0.4751 | 29993 | 19236 | 180 | 0.6025 | 2404 | 3084 | 180 | 0.6827 | 215 | 421 | 176 | 0.7506 | 17 | 48 |
| 240 | 240 | 0.4025 | 240243 | 42272 | 240 | 0.3443 | 34654 | 10363 | 240 | 0.4237 | 3241 | 1758 | 240 | 0.4754 | 333 | 245 | 240 | 0.5718 | 28 | 34 |
| 300 | 300 | 0.3112 | 247696 | 19017 | 300 | 0.2419 | 37292 | 5139 | 300 | 0.2847 | 3741 | 880 | 300 | 0.3491 | 393 | 148 | 291 | 0.4178 | 36 | 20 |
| 360 | 360 | 0.2344 | 251262 | 7792 | 360 | 0.1632 | 38655 | 2184 | 360 | 0.1769 | 4020 | 370 | 360 | 0.2483 | 429 | 73 | 324 | 0.3329 | 40 | 12 |
| 420 | 420 | 0.1720 | 252775 | 2654 | 420 | 0.1054 | 39268 | 783 | 418 | 0.0990 | 4146 | 118 | 419 | 0.1339 | 456 | 24 | 388 | 0.2747 | 42 | 8 |
| 480 | 480 | 0.1143 | 253245 | 144 | 477 | 0.0595 | 39482 | 54 | 478 | 0.0461 | 4187 | 11 | 472 | 0.0507 | 464 | 2 | 474 | 0.1373 | 45 | 1 |
| 500 | 491 |  | 253257 | 0 | 484 |  | 39484 | 0 | 485 |  | 4189 | 0 | 472 |  | 464 | 0 | 474 |  | 45 | 0 |

**B**.

|  | No subsequent tumor | | | | One subsequent tumor | | | | Two subsequent tumors | | | | Three subsequent tumors | | | | Four or more subsequent tumors | | | |
| --- | --- | --- | --- | --- | --- | --- | --- | --- | --- | --- | --- | --- | --- | --- | --- | --- | --- | --- | --- | --- |
| Timelist (months) | Event Months | Survival probability | Number Failed | Number Left | Event Months | Survival probability | Number Failed | Number Left | Event Months | Survival probability | Number Failed | Number Left | Event Months | Survival probability | Number Failed | Number Left | Event Months | Survival probability | Event Months | Survival probability |
| 24 | 24 | 0.9212 | 43016 | 462339 | 24 | 0.9770 | 863 | 36337 | 24 | 0.9959 | 14 | 3384 | 20 | 0.9971 | 1 | 343 | 0 | 1.0000 | 0 | 33 |
| 60 | 60 | 0.8213 | 87842 | 315685 | 60 | 0.8975 | 3733 | 31117 | 60 | 0.9662 | 114 | 3214 | 60 | 0.9796 | 7 | 334 | 57 | 0.9697 | 1 | 32 |
| 120 | 120 | 0.7290 | 116334 | 160748 | 120 | 0.7840 | 7291 | 21184 | 120 | 0.8874 | 363 | 2592 | 119 | 0.9369 | 21 | 296 | 57 | 0.9697 | 1 | 31 |
| 180 | 180 | 0.6682 | 125883 | 66230 | 180 | 0.6911 | 9338 | 11581 | 180 | 0.7859 | 621 | 1660 | 172 | 0.8569 | 44 | 220 | 176 | 0.8542 | 4 | 21 |
| 240 | 240 | 0.6202 | 129462 | 31343 | 240 | 0.6185 | 10326 | 6381 | 240 | 0.6914 | 792 | 1000 | 240 | 0.7430 | 69 | 137 | 240 | 0.8067 | 5 | 17 |
| 300 | 300 | 0.5818 | 130856 | 14147 | 300 | 0.5578 | 10815 | 3306 | 300 | 0.6003 | 895 | 519 | 293 | 0.6518 | 84 | 85 | 240 | 0.8067 | 5 | 11 |
| 360 | 360 | 0.5523 | 131358 | 5986 | 360 | 0.5017 | 11057 | 1472 | 359 | 0.5184 | 947 | 236 | 351 | 0.6092 | 88 | 42 | 240 | 0.8067 | 5 | 7 |
| 420 | 420 | 0.5318 | 131514 | 2205 | 419 | 0.4587 | 11149 | 592 | 418 | 0.4125 | 979 | 78 | 401 | 0.5505 | 91 | 17 | 240 | 0.8067 | 5 | 5 |
| 480 | 467 | 0.5181 | 131546 | 133 | 477 | 0.4231 | 11174 | 52 | 446 | 0.3907 | 982 | 9 | 430 | 0.5082 | 92 | 2 | 240 | 0.8067 | 5 | 1 |
| 500 | 488 |  | 131547 | 0 | 477 |  | 11174 | 0 | 446 |  | 982 | 0 | 430 |  | 92 | 0 | 240 |  | 5 | 0 |

eTable 4. Survival rate information for cancer-specific mortality stratified by with or without secondary malignancies.

|  | No subsequent malignancy | | | | With subsequent malignancy | | | |
| --- | --- | --- | --- | --- | --- | --- | --- | --- |
| Timelist (months) | Event Months | Survival probability | Number Failed | Number Left | Event Months | Survival probability | Number Failed | Number Left |
| 24 | 24 | 0.9212 | 43016 | 462339 | 24 | 0.9787 | 878 | 40097 |
| 60 | 60 | 0.8213 | 87842 | 315685 | 60 | 0.9040 | 3855 | 34697 |
| 120 | 120 | 0.7290 | 116334 | 160748 | 120 | 0.7946 | 7676 | 24103 |
| 180 | 180 | 0.6682 | 125883 | 66230 | 180 | 0.7011 | 10007 | 13482 |
| 240 | 240 | 0.6202 | 129462 | 31343 | 240 | 0.6258 | 11192 | 7535 |
| 300 | 300 | 0.5818 | 130856 | 14147 | 300 | 0.5614 | 11799 | 3921 |
| 360 | 360 | 0.5523 | 131358 | 5986 | 360 | 0.5029 | 12097 | 1757 |
| 420 | 420 | 0.5318 | 131514 | 2205 | 419 | 0.4523 | 12224 | 692 |
| 480 | 467 | 0.5181 | 131546 | 133 | 477 | 0.4187 | 12253 | 64 |
| 500 | 488 |  | 131547 | 0 | 477 |  | 12253 | 0 |

eTable 5. Survival rate information for cancer-specific mortality stratified by first breast cancer diagnosis decades.

|  | 1973-1984 | | | | 1985-1994 | | | | 1995-2004 | | | | 2005-2014 | | | |
| --- | --- | --- | --- | --- | --- | --- | --- | --- | --- | --- | --- | --- | --- | --- | --- | --- |
| Timelist (months) | Event Months | Survival probability | Number Failed | Number Left | Event Months | Survival probability | Number Failed | Number Left | Event Months | Survival probability | Number Failed | Number Left | Event Months | Survival probability | Number Failed | Number Left |
| 24 | 24 | 0.8227 | 6661 | 30873 | 24 | 0.8833 | 7255 | 54882 | 24 | 0.9263 | 13316 | 167102 | 24 | 0.9461 | 16662 | 249579 |
| 60 | 60 | 0.5883 | 15444 | 21992 | 60 | 0.7255 | 17049 | 44940 | 60 | 0.8403 | 28789 | 150715 | 60 | 0.8837 | 30415 | 132735 |
| 108 | 108 | 0.4303 | 21334 | 15970 | 108 | 0.6183 | 23675 | 38153 | 108 | 0.7767 | 40137 | 137287 | 108 | 0.8329 | 34920 | 20440 |
| 120 | 120 | 0.4053 | 22261 | 15023 | 120 | 0.6011 | 24797 | 37036 | 120 | 0.7662 | 41993 | 132792 | 117 |  | 35019 | 0 |
| 180 | 180 | 0.3212 | 25363 | 11777 | 180 | 0.5352 | 28782 | 32591 | 180 | 0.7230 | 46726 | 35344 | 117 |  | 35019 | 0 |
| 240 | 240 | 0.2773 | 26964 | 10012 | 240 | 0.4975 | 31159 | 28866 | 237 |  | 47512 | 0 | 117 |  | 35019 | 0 |
| 300 | 300 | 0.2499 | 27944 | 8806 | 300 | 0.4688 | 32180 | 9262 | 237 |  | 47512 | 0 | 117 |  | 35019 | 0 |
| 360 | 360 | 0.2317 | 28575 | 7743 | 356 |  | 32349 | 0 | 237 |  | 47512 | 0 | 117 |  | 35019 | 0 |
| 420 | 420 | 0.2196 | 28858 | 2897 | 356 |  | 32349 | 0 | 237 |  | 47512 | 0 | 117 |  | 35019 | 0 |
| 480 | 477 | 0.2110 | 28919 | 197 | 356 |  | 32349 | 0 | 237 |  | 47512 | 0 | 117 |  | 35019 | 0 |
| 500 | 488 |  | 28920 | 0 | 356 |  | 32349 | 0 | 237 |  | 47512 | 0 | 117 |  | 35019 | 0 |

eTable 6. Survival rate information for cancer-specific mortality in 2005-2014 stratified by with or without secondary malignancies.

|  | 2005-2014 without subsequent malignancies | | | | 2005-2014 with subsequent malignancies | | | |
| --- | --- | --- | --- | --- | --- | --- | --- | --- |
| Timelist (months) | Event Months | Survival probability | Number Failed | Number Left | Event Months | Survival probability | Number Failed | Number Left |
| 12 | 12 | 0.9721 | 8949 | 285575 | 12 | 0.9948 | 58 | 10993 |
| 24 | 24 | 0.9452 | 16337 | 239248 | 24 | 0.9702 | 325 | 10331 |
| 36 | 36 | 0.9203 | 22210 | 196898 | 36 | 0.9414 | 624 | 9415 |
| 48 | 48 | 0.8989 | 26438 | 160014 | 48 | 0.9104 | 923 | 8339 |
| 60 | 60 | 0.8817 | 29229 | 125670 | 60 | 0.8802 | 1186 | 7065 |
| 72 | 72 | 0.8671 | 31101 | 94941 | 72 | 0.8522 | 1395 | 5741 |
| 84 | 84 | 0.8548 | 32267 | 66601 | 84 | 0.8280 | 1544 | 4382 |
| 96 | 96 | 0.8452 | 32895 | 41709 | 96 | 0.8078 | 1637 | 2881 |
| 108 | 108 | 0.8362 | 33228 | 19046 | 108 | 0.7878 | 1692 | 1394 |
| 120 | 117 |  | 33310 | 0 | 117 |  | 1709 | 0 |

eTable 7. Survival rate information for cancer-specific mortality stratified by subsequent malignancy sites.

|  | Breast | | | | Lung / Bronchus | | | | Ovary / Uteri | | | | Cecum / Colon / Rectum / Anus | | | | Lymphoma / Leukemia | | | | Thyroid | | | |
| --- | --- | --- | --- | --- | --- | --- | --- | --- | --- | --- | --- | --- | --- | --- | --- | --- | --- | --- | --- | --- | --- | --- | --- | --- |
| Timelist (months) | Event Months | Survival probability | Number Failed | Number Left | Event Months | Survival probability | Number Failed | Number Left | Event Months | Survival probability | Number Failed | Number Left | Event Months | Survival probability | Number Failed | Number Left | Event Months | Survival probability | Event Months | Survival probability | Event Months | Survival probability | Event Months | Survival probability |
| 24 | 24 | 0.8517 | 2478 | 12965 | 24 | 0.6399 | 1080 | 1487 | 24 | 0.8949 | 436 | 3257 | 24 | 0.8937 | 331 | 2421 | 24 | 0.9664 | 39 | 992 | 24 | 0.8828 | 208 | 1271 |
| 60 | 60 | 0.7105 | 4394 | 8136 | 60 | 0.4946 | 1352 | 684 | 60 | 0.8083 | 709 | 2110 | 60 | 0.8055 | 537 | 1536 | 57 | 0.9157 | 81 | 565 | 60 | 0.8180 | 285 | 762 |
| 120 | 120 | 0.5881 | 5513 | 3787 | 120 | 0.3954 | 1447 | 225 | 120 | 0.7317 | 867 | 1011 | 120 | 0.7072 | 679 | 641 | 120 | 0.8747 | 99 | 206 | 119 | 0.7421 | 333 | 275 |
| 180 | 180 | 0.5100 | 5897 | 1762 | 171 | 0.3275 | 1474 | 73 | 178 | 0.6801 | 917 | 433 | 180 | 0.6248 | 729 | 244 | 160 | 0.8431 | 104 | 82 | 180 | 0.6797 | 348 | 85 |
| 240 | 239 | 0.4610 | 6020 | 806 | 237 | 0.2636 | 1484 | 26 | 236 | 0.6149 | 944 | 146 | 233 | 0.5879 | 739 | 94 | 211 | 0.7909 | 108 | 35 | 208 | 0.5983 | 354 | 14 |
| 300 | 295 | 0.4236 | 6068 | 311 | 237 | 0.2471 | 1485 | 6 | 274 | 0.5841 | 949 | 51 | 293 | 0.5023 | 748 | 31 | 298 | 0.7314 | 110 | 21 | 208 | 0.5983 | 354 | 5 |
| 360 | 357 | 0.3909 | 6083 | 108 | 257 | 0.2471 | 1485 | 3 | 334 | 0.5375 | 952 | 14 | 293 | 0.5023 | 748 | 8 | 298 | 0.7314 | 110 | 9 | 208 |  | 354 | 2 |
| 420 | 417 | 0.3585 | 6087 | 21 | 257 | 0.2471 | 1485 | 1 | 334 | 0.5375 | 952 | 3 | 293 | 0.5023 | 748 | 1 | 383 | 0.6269 | 111 | 2 | 208 |  | 354 | 0 |
| 480 | 417 |  | 6087 | 0 | 257 |  | 1485 | 0 | 334 |  | 952 | 0 | 293 |  | 748 | 0 | 383 |  | 111 | 0 | 208 |  | 354 | 0 |
| 500 | 417 |  | 6087 | 0 | 257 |  | 1485 | 0 | 334 |  | 952 | 0 | 293 |  | 748 | 0 | 383 |  | 111 | 0 | 208 |  | 354 | 0 |
